# A cross-sectional study of mental health-, posttraumatic stress symptoms and post exposure changes in Norwegian ambulance personnel

**DOI:** 10.1186/s13049-021-00991-2

**Published:** 2022-01-11

**Authors:** Bjørn Ole Reid, Lars Eide Næss-Pleym, Karin Elvenes Bakkelund, Jostein Dale, Oddvar Uleberg, Andreas Espetvedt Nordstrand

**Affiliations:** 1 Department of Emergency Medicine and Prehospital Services, St. Olav’s Hospital, Prinsesse Kristinas gate 3, AHL, 7030 Trondheim, Norway; 2 Joint Medical Services, Norwegian Armed Forces, Sessvollmoen, Norway; 3 Department of Circulation and Medical Imaging, NTNU Norwegian University of Science and Technology, Trondheim, Norway; 4 Department of Research and Development, Norwegian Air Ambulance Foundation, Drøbak, Norway; 5 Department of Psychology, NTNU Norwegian University of Science and Technology, Trondheim, Norway

## Abstract

**Background:**

Posttraumatic stress disorder (PTSD) has been shown to be elevated among first responders (Emergency Medical Services, fire service, police force) compared to the general population. Examining the prevalence of mental health issues in a work force with an elevated occupational risk is fundamental towards ensuring their wellbeing and implementing safeguard measures. The goal of this study is therefore to report the prevalence of depression, anxiety, posttraumatic development, and PTSD in Norwegian ambulance personnel.

**Methods:**

This study is a cross-sectional, anonymous, web-based survey (Questback®), performed among operative personnel employed in the Emergency Medical Services in the Regional Health Trust of Central Norway between 18. February and 9. April 2021. The study was sent to 1052 eligible participants. Questions reported demographic data, a traumatic events exposure index, Patient Health Questionnaire-9 (Depression), Generalized Anxiety Disorder-7 scale, Posttraumatic symptom scale (PTSD) and Posttraumatic change scale.

**Results:**

The response rate in this study was 45.5% (n = 479/1052). The mean age of respondents was 37.1 years (std. 11.1) and 52.8% (n = 253) were male. Of the respondents, 80.6% (n = 386) were married or had a partner, and 91.6% (n = 439) reported having access to a peer support programme, with 34.9% (n = 167) reporting that they had utilized peer support. In this study, 5% (n = 24) showed a prevalence of manifest posttraumatic stress disorder symptoms, while 8.6% (n = 41) reported moderate to severe depression and 2.9% (n = 14) presented moderate to severe symptoms of general anxiety. Of the respondents, 77.2% (n = 370) reported personal growth because of their work experiences.

**Conclusions:**

This study indicates that Norwegian ambulance personnel report a prevalence of posttraumatic stress symptoms and depression, which is slightly higher for men, and lower for the female proportion in this study, when compared to an adult Norwegian population. The vast majority of respondents reported personal growth because of their work experience, and both the degree of peer support and having a partner seem to influence levels of posttraumatic stress and -development.

**Supplementary Information:**

The online version contains supplementary material available at 10.1186/s13049-021-00991-2.

## Background

As part of their regular work load emergency service personnel are exposed to an array of potentially traumatic events, which may influence their physical or psychological wellbeing [1]. Such stressors may include acts of violence, traffic related accidents, physical exertion, exposure to the elements and risk of transmission of diseases [2–4]. The latter has been accentuated by the COVID-19 pandemic [5]. Exposure to traumatic events entail increased risk of developing posttraumatic stress symptoms, as well as other forms of psychological distress such as depression and anxiety [6]. Accordingly, Posttraumatic Stress Disorder (PTSD) has been shown to be elevated among first responders (Emergency Medical Services (EMS), fire service, police force) compared to the general population [7, 8].

Studies indicate that EMS personnel have the highest rate of PTSD among these first responder service branches [9]. This may be reflected in the characteristics of several health-related professions working in the pre- or in-hospital arena. Doctors, nurses and paramedics employed in emergency medicine all report high exposure rates to traumatic and stressful events, which may negatively affect their sense of wellbeing and increase levels of psychological distress [10].

The precipitating events and criterion of PTSD are defined by the fifth edition of the Diagnostic and Statistical Manual of Mental Disorders (DSM-V) [11]. Specifically, the DSM-V formulates traumatic exposure as the direct or witnessed experience of actual or threatened death, serious injury or sexual violation, or first-hand repeated or extreme exposure to aversive details of the traumatic event. The behavioural symptoms of PTSD are described as re-experiencing, avoidance, negative cognitions and mood, and arousal. Since the establishment of the PTSD diagnosis, there has been an increasing emphasis on the potential for negative mental health outcomes after exposure to traumatic events [12]. However, chronic PTSD is by no means the most likely consequence of experiencing trauma. Most people quickly regain normal function after exposure to traumatic stress, a phenomenon commonly labelled resilience [13]. Moreover, even among those who suffer from trauma related distress, it is not uncommon for the traumatic experience to also instigate a trajectory of positive psychological development, often referred to as posttraumatic growth (PTG) [14].

Examining the prevalence of mental health issues in a work force with an elevated occupational risk is fundamental towards ensuring their wellbeing and implementing safeguard measures. The goal of this study is therefore to report the prevalence of depression, anxiety and PTSD in Norwegian ambulance personnel. Due to recent studies showing that traumatic exposure may also impact both negatively and positively on personal development, we also measured the degree of reported posttraumatic growth or deprecation as a consequence of exposure to work-related events [15].

## Methods

### Study setting

The Regional Health Trust of Central Norway is one of four regional health trusts. It funds and manages 65 EMS stations in a geographical diverse area with mountains, fjords, Atlantic coastline, towns and cities, covering 56,558 square kilometres. In 2019 the region had a population of 731,480, and the EMS performed 94,250 missions, of which 38.1% were emergency red responses.

Personnel employed in the Norwegian EMS are primarily emergency medical technicians (EMT). The paramedic title in Norway largely belongs to EMT’s with additional education, but a new Bachelor paramedic program is currently in progress. In addition, qualified nurses also work in EMS. Normally, there is a crew of two EMT’s working on an ambulance. They are dispatched and coordinated by the Emergency Medical dispatch centre, of which there are three in this region. Identical peer-to-peer support programs have been implemented in two of the three health trusts (Nord- and Sør-Trøndelag) involved in the study. This entails an education of selected co-workers in defusing and debriefing techniques based on one-to-one meetings. Criteria for activation are similar to the events described in the Traumatic events exposure index (Table 2) utilized in this study. In the third health trust (Møre and Romsdal), support relies on the involvement of a superior, who is responsible for the further process.

### Study design

This study is a cross-sectional, anonymous, web-based survey (Questback®), performed among operative personnel employed in the EMS in the Regional Health Trust of Central Norway between 18. February and 9. April 2021. The leaders of all the responsible departments and ambulance stations in the Health Trusts were contacted for confirmation to participate, and for updated electronic mail address lists for those eligible to take part in the study.

The study was sent to 1052 eligible participants, 558 male and 494 female. Participation was voluntary, and required consent from participants. During the study period, two reminders were sent to non-responders, by the same electronic mail format.

The survey consisted of questions including:

1. Demographical data (age, gender, full-time/part-time, length of work experience, professional background (EMT/paramedic/nurse/trainee), cohabitation, peer support access).
2. Traumatic events exposure index, 10 items (Developed for current study. Identification of traumatic events based on peer support programme for Health Trust Sør Trøndelag EMS). The participant reports exposure to defined traumatic events from the working environment during the last 12 months. In addition, the participant is asked to report if the event was perceived as a traumatic experience.
3. PHQ-9 (Patient Health Questionnaire) is a tool specific to depression, and scores each of the nine DSM-IV criteria based on the mood modules from the original PRIME-MD (Primary Care Evaluation of Mental Disorders [16, 17]. Contains nine items, score for each item 0–3, score range 0–27. In clinical studies, lowest score of 10 is used as a cut-off value for moderately severe depression.Cronbach’s α = 0.847 (Mean = 4.3, SD = 3.7).
4. GAD-7 (Generalized Anxiety Disorder) scale. Contains seven items, score for each item 0–3, score range 0–21. Score 5–9 indicates mild anxiety, 10–14 moderate anxiety and 15–21 severe anxiety [18]. In this study the lowest score of 10 is used as a cut-off value for moderately severe anxiety.
  Cronbach’s α = 0.815 (Mean = 3.0, SD = 2.8).
5. PTCS (Posttraumatic change scale) contains 12 items. It investigates three dimensions: self-confidence, interpersonal involvement including social adaptability, and awareness, Developed and validated by the Norwegian Joint Medical Services [15]. Scores indicate PTG (posttraumatic growth)/PTD (posttraumatic deprecation)/no-change. Items are rated on a 5-point Likert scale (1–5). Mean score of 3 indicates no-change, in this study set at mean values between, but not including 2.99 and 3.1. Mean score above 3 indicates PTG, in this study defined from 3.1to 5, and mean score below 3 indicates PTD, in this study values from 1 to 2.99.
  Cronbach’s α = 0.885 (Sum mean = 41.1, SD = 6.2).
6. PTSS (Posttraumatic symptom scale) contains 10 items measuring posttraumatic symptoms during the last 7 days. It consists of a 7-point Likert scale (1–7) with possible total score range 10–70. In this study the total sum score ≥ 35 is considered the cut-off threshold for probable PTSD. It was developed and validated for a Norwegian population [19].
  Cronbach’s α = 0.901 (Mean = 17.3, SD = 8.8).

All questions were submitted in Norwegian language. The Additional file 1 contains English versions of the scales applied in this study.

### Statistical analysis

Data was analyzed using the Statistical Package for the Social Sciences (IBM® SPSS® Statistics, Version 27 IBM Corp, Armonk, NY, USA). Statistical data is presented as frequencies, percentages, average with mean and standard deviation (SD), median with interquartile range (IQR), where appropriate. Statistical comparisons between the demographic variables (independent) and dependent test variables (PHQ-9, GAD-7, PTSS, PTCS, Traumatic events exposure index) were performed using Pearson’s Chi square test or Fischer’s test, where applicable. Independent Sample t-test was applied to compare means of age. A simple linear regression analysis was performed to estimate strength of association between PHQ-9, GAD-7 and PTSS. Statistical significance was defined by p < 0.05.

Internal consistency of the different scales/tests applied in this study was measured utilizing Crombach’s alpha, which is reported in the description of the appropriate scale in this methods section.

## Results

The study was answered by 500 (47.5%) recipients, including 21 (2%) who declined to take part. Ultimately, 479 (45.5%) consented to, and completed the questionnaire, and are therefore included in the study. The mean age of respondents was 37.1 years (std. 11.1), 52.8% (n = 253) were male (mean age 39.6 (std. 11.2)) and 47.2% (n = 226) were female (mean age 34.4 (std.10.4)). The median number of years of work experience in EMS was 12 (IQR 12–19), and reported education level was as follows: student/trainee 8.4% (n = 40), EMT 46.1% (n = 221), nurse 21.1% (n = 101), paramedic 24.4% (n = 117). Of the respondents, 80.6% (n = 386) were married or had a partner, and 91.6% (n = 439) reported having access to a peer support programme, with 34.9% (n = 167) reporting that they had utilized peer support.

The median score in the study population for depression (PHQ-9) was 3 (IQR 2–6), anxiety (GAD-7) was 2 (IQR 1–4) and posttraumatic stress (PTSS) was 14 (IQR 11–20). Further results on anxiety, depression and posttraumatic stress symptoms in the study population are reported in Table 1.

**Table 1:** Depression, anxiety and posttraumatic stress symptoms in study population

Psychological test	Depression (PHQ-9 ≥ 10) n (%)	Anxiety (GAD-7 ≥ 10) n (%)	Posttraumatic symptom scale (PTSS ≥ 35) n (%)	Posttraumatic symptom scale Median (IQR)
Total (n = 479)	41 (8.6)	14 (2.9)	24 (5)	14 (11–20)
Male (n = 253)	24 (9.5)	6 (2.4)	12 (4.7)	14 (10–18)
Female (n = 226)	17 (7.5)	8 (3.5)	12 (5.3)	15 (11–22.25)
Married/partner (n = 386)	29 (7.5)	9 (2.3)	15 (3.9)	14 (11–20)
Unmarried/no partner (n = 93)	12 (12.9)	5 (5.4)	9 ( 9.7)*	15 (12–22)
Peer Support access (n = 439)	33 (7.5)	11 (2.5)	19 (4.3)	14 (11–19)
No access peer support (n = 40)	8 (20)*	3 (7.5)	5 (12.5)*	17.5 (12.25–25.75)
Utilized peer support (n = 167)	15 (9)	4 (2.4)	8 (4.8)	15 (12–20)
Peer support not utilized (n = 312)	26 (8.3)	10 (3.2)	16 (5.1)	14 (10–20.75)
Student (n = 40)	6 (0.15)	2 (5)	2 (5)	15.5 (12–24.75)
EMT (n = 221)	24 (10.9)	10 (4.5)	13 (5.9)	15 (11–21.5)
Nurse (n = 101)	5 (5)	2 (0.2)	2 (0.2)	14 (10–18)
Paramedic (n = 117)	6 (5.1)	0 (0)	3 (2.6)	14 (11–20)

*PHQ* Patient Health Questionnaire, *GAD* Generalized Anxiety Disorder scale, *PTSS* posttraumatic symptom scale, IQR inter quartile range

*Fisher Exact Test p < 0.05

The threshold for manifest posttraumatic stress disorder symptoms in this self-reporting study was defined as a score ≥ 35 on the PTSS. In this study, n = 24 (5%) attained this score (mean 34 years (std. 1.8)) (Table 2). Women in this study with manifest post traumatic stress symptoms had a statistically lower age (mean age = 31.6 (std. 8.1)) compared to men (mean age = 36.5 (std. 9.1)) (p < 0.05).

**Table 2:** Exposure to potentially emotionally traumatic events in working environment last 12 months and corresponding Posttraumatic symptom scale score ≥ 35

Exposure group (Traumatic events exposure index)	Total n	PTSS ≥ 35 n (% of total)	Significance	Subjectively traumatic experience n	PTSS ≥ 35 n (% of traumatic experience)
Total study population	479	24 (5)	–	–	–
Death of young patient < 30 years	124	5 (4)	No	32	2 (6.3)
Serious illness/injury in young patient < 30	275	16 (5.8)	No	27	3 (11.1)
Patient with serious injuries	355	18 (5.1)	No	20	3 (15)
Suicide (including attempt)	242	12 (5)	No	32	4 (12.5)
Death generally	416	23 (5.5)	No	34	3 (8.8)
Inability to help critically ill/injured patient	96	9 (9.4)	No	26	7 (26.9)
Threats, violence or aggression towards you or colleague	267	17 (6.4)	No	53	7 (13.2)
Threats, violence or aggression towards patients	224	10 (4.5)	No	15	4 (26.7)
Accident in ambulance	45	6 (13.3)	Yes*	8	1 (12.5)
Diagnostic-/treatmentdeviation causing patient injury/-deterioration	36	6 (16.7)	Yes*	9	1 (11.1)

An accident is defined as an unplanned event that has caused equipment damage or human injury requiring medical attention (This does not relate to patient treatment)

*PTSS* posttraumatic symptom scale, *Sig* significance

*Fisher Exact Test p < 0.05

A simple linear regression was performed to predict the relationship between PTSS as dependent value, with PHQ-9 (anxiety) or GAD-7 (depression) as independent values. The assumption of normality and linearity was not violated in these cases. In both cases we found a positive relationship (PHQ-9: b = 0.802, t = 29.28, p < 0.001/GAD-7: b = 0.796, t = 28.7, p < 0.001).

The Posttraumatic change scale was applied in this study to determine perceived emotional growth, deprecation or no-growth as a self-reported consequence of work experiences in EMS (Table 3). Mean scores above three indicate growth. There was no statistical significant difference regarding post traumatic growth between the professional backgrounds working in the EMS.

**Table 3:** Posttraumatic change scale

Post traumatic change scale (PTCS)	Clinical threshold	Study population (n = 479) n (%)	Male (n = 253) n (%)	Female (n = 226) n (%)	Married/Partner (n = 386) n (%)	Unmarried/no partner (n = 93) n (%)	Peer support access (n = 439) n (%)	No peer support access n = (40) n (%)	PTSS ≥ 35 (n = 24) n (%)	PTSS < 35 (n = 455) n (%)
PTCS (growth/PTG)	Mean > 3	351 (73.2)	183 (72.3)	168 (74.3)	296 (76.7)	55 (59.1)	331 (75.4)	20 (50)	7 (29.2)	344 (75.6)
PTCS (no change)	Mean = 3	52 (10.9)	32 (12.7)	20 (8.9)	36 (9.3)	16 (17.2)	47 (10.7)	5 (12.5)	3 (12.5)	49 (10.8)
PTCS (deprecation/PTD)	Mean < 3	76 (15.9)	38 (15)	38 (16.8)	54 (14)	22 (23.7)*	61 (13.9)	15 (37.5)*	14 (58.3)**	62 (13.6)

*PTG* posttraumatic growth, *PTD* posttraumatic deprecation

*Fisher Exact Test p < 0.05; **Pearson Chi-Square p < 0.05

Of the 76 respondents who reported deprecation (Table 3), 33 (43.4%) had made use of a peer support program, a higher proportion than the respondents with PTSS ≥ 35, where 8 (33%) reported the same. Similarly, of the 93 respondents who were unmarried/no partner, 41 (44.1%) reported using the accessible program.

## Discussion

In this study, Norwegian ambulance personnel showed a prevalence of manifest posttraumatic stress disorder symptoms of 5%, while 8.6% reported moderate to severe depression and 2.9% presented moderate to severe symptoms of general anxiety. Over half of the respondents had experienced threats or aggression towards themselves or colleagues in the past 12 months. We observed increased posttraumatic stress symptoms in workers who were unmarried/no partner, or those reporting no access to peer support. Moreover, exposure to ambulance accidents or feelings of inadequacy towards patients regarding treatment errors was also associated with increased reporting of posttraumatic stress symptoms in this study. The vast majority of respondents reported personal growth as a consequence of their work experiences.

The reported levels of posttraumatic symptoms in this study were lower than recent systematic reviews of PTSD in ambulance personnel, where the prevalence was quoted at 10.2% and 11% respectively [9, 20]. However, the current results are more in alignment with other European surveys conducted in cohorts that may be culturally similar to Norwegian EMS personnel, with one German study reporting a positive PTSD screening of 5.4%, and in Switzerland with 4.3% [21, 22].

The current study found that PTSD among EMS workers was more common among men than what is generally reported in the Norwegian general adult male population. A recent study of Norwegian adults described a point prevalence of PTSD of 3.8% (men) and 8.5% (women), while the current study reported 4.7% men and 5.3% women [23]. Of note, our study showed no significant difference in PTSD prevalence with regards to biological sex, professional background, nor percentage of employment. This result deviates from findings in the Norwegian general population, and the discrepancy merits further studies.

The relative low prevalence of reported PTSD in this study may have several reasons. First, EMS in Norway has become increasingly professionalized, with focus on selection, training and further education. Almost half of respondents in this study were either qualified nurses or paramedics, and more than 80% were in full time positions. Studies among fire fighters have shown increased PTSD among part-time versus full-time employees [24]. There has also been increasing focus on mental wellbeing within EMS, furthering knowledge and possibly lowering the threshold to seek help. In our study population, 47.2% of participants were women. A study of Norwegian ambulance personnel performed in 2005 included 23.2% women in comparison [25]. It is possible, that an increasing female proportion in Norwegian EMS has contributed to a culture of more openness and acceptance, as opposed to more male dominated work cultures [26]. Studies have also shown, that women in the military or working as first responders show no higher rates of PTSD compared to men. This can possibly be explained by selection [9, 21, 27, 28]. Women in this study had a lower average age compared to men, and this may also explain the lower age of women with positive PTSD screening found in this study.

A recent Norwegian study of the general population showed a prevalence of self-diagnosed current depression at 8.1%, higher for women (9.8%) than men (6.1%), and another similar Norwegian study reported anxiety at 6.6% [29, 30]. This was comparable to the current study, where 8.6% reported depression (men 9.4%/women 7.5%), whereas anxiety prevalence was considerably lower, at 2.9%. We found a positive correlation between severity of depression and anxiety, and increased PTSS scores. This finding is important, because it indicates that mental health issues and PTSS are associated with each other, although this study does not clarify a cause and effect relationship between the two. A study of Norwegian ambulance personnel by Sterud et al. reported serious suicidal ideation in 10.4% of the study population, describing a strong relationship with depression symptoms [31]. In addition they reported that more than half of this group had not sought professional help, emphasizing the importance of appropriate support and guidance within the service.

The implementation of preventative measures in the work environment may improve the mental wellbeing of employees [32]. In this study, more than 90% of participants reported having access to a peer support programme, with over a third having utilized it one time or another. One health trust in the region endorses a more superior-to-peer intervention. This may explain why over 8% reported no access to peer support, although this study does not differentiate between the health trusts. Another reason could be lack of information regarding the programmes. However, successful peer support initiatives have been characterized as involving strict peer-to-peer interventions with same-levelled individuals, as is the concept in the remaining two health trusts in the region [33]. In this study there was an association between increased post traumatic stress symptoms and reporting having no access to peer support, and the same applied to respondents who were not in a marital-/partner relationship. Likewise, we observed similar associations between post traumatic deprecation (negative emotional growth) and respondents with no partners or lacking peer support, emphasizing the positive effect of social support on mental wellbeing, mitigating PTSD and facilitating post traumatic growth, as reported in other studies [34, 35].

This study indicates that Norwegian EMS personnel are regularly exposed to potentially traumatic events. Traditionally, traumatic stress has been understood as relating to life-threatening events and subsequent fear dysregulation, and it may be more intuitive to associate exposure to violent events and threats with increased stress, which we also observed in this survey. However, in many traumatic situations, peritraumatic fear may not be present, and a physical threat may not be the most stressful part of the incident [36, 37]. Accordingly, recent studies have distinguished between danger-based and non-danger-based stressors [38]. In the context of the current study, this may help explain why perceptions of inadequacy and even shame, also committing errors, may lead to increased posttraumatic stress reactions.

Resilience in this context can be described as the ability of an individual to adjust to challenges and influences which potentially may be psychological harmful [39]. That more than 90% of our sample did not report any mental health complaints, despite the substantial degree of exposure to traumatic stressors, indicates high levels of resilience in the study cohort. This may be due to training and preparedness for the most commonly encountered stressors, and the efficacy of existing peer support programs. Moreover, we included the Posttraumatic change scale (PTCS) in this survey, and over three quarters of participants reported experiencing growth after exposure to experiences. The concept of “posttraumatic growth” encompasses more than a return to normal function after a period of distress, but indicates “*positive psychological changes experienced as a result of the struggle with highly challenging life circumstances*” [40]. This model provides a positive outlook on how personnel working in demanding professions can learn and develop emotionally from distressing experiences, even if such events precipitate a period of psychological suffering [15, 41].

This study has several limitations. The response rate was 46%, which means that findings should be interpreted with caution. Missing answers could definitely influence results, and there may be a risk that the most troubled by traumatic experiences did not answer our survey. However, response rates have been described as a typical challenge in studies of related occupational groups [20]. In similar studies of ambulance personnel, response rates have varied greatly, illustrated by the following reported rates in studies: 5%, 7%, 26%, 32%, 41%, 72% and 77% [20, 21, 31, 41–44]. Regarding gender differences, the ratio of those invited to participate was 53% men and 47% women, virtually identical to the response ratio. The study incorporated three different health trusts, with varying geography and both urban and rural demographics, supporting the applicability of the findings as being representative of a Norwegian ambulance population. Due to the nature of the study, a self-reported survey carries the risk of both reporting bias and recall bias. Reporting bias may however be mitigated by the use of questions which are not open-ended, and the anonymity of respondents.

Validated tests were applied to measure posttraumatic stress, posttraumatic growth, depression and anxiety. This study can only give an indication of the prevalence of these serious conditions, and self-reported questionnaires cannot compare to diagnostic interviews in this respect [45]. In addition, this survey was performed among current operative personnel. Therefore, we have no data on personnel on prolonged leave, those who have retired or left the service, or personnel now working in administrative positions, irrespective of the reason for changing their employment status. It is possible to assume, that personnel who are struggling to cope in an operative setting, whatever the reason, may choose to move on, or work in a different capacity, thereby distorting the prevalence of certain conditions in our data.

## Conclusion

This study indicates that Norwegian ambulance personnel report a prevalence of posttraumatic stress symptoms and depression, which is slightly higher for men, and lower for the female proportion in this study, when compared to an adult Norwegian population. We observed an association of increased posttraumatic stress symptoms in workers who were unmarried/had no partner, in those reporting no access to peer support, or who experienced feelings of inadequacy towards patients, including those exposed to ambulance accidents.

The vast majority of respondents reported personal growth as a consequence of their work experience, and both the degree of peer support and having a partner seem to influence levels of posttraumatic growth. On an institutional level, we therefore recommend having robust, accessible and widely advertised peer support programs in place, to mitigate the negative effects of an apparently demanding profession, and would advocate for similar studies among other prehospital first responders, for instance the air ambulance or fire department.

## Supplementary Information

**Additional file 1**. English version of inex and scales.

**Additional file 2**. Study invitation and consent to participation.

## Data Availability

The datasets used during the current study may be made available from the corresponding author upon reasonable request.

## Abbreviations

PTSD: Posttraumatic stress disorder
EMS: Emergency medical services
DSM-V: Diagnostic and Statistical Manual of Mental Disorders 5th edition
PTG: Posttraumatic growth
PTD: Posttraumatic deprecation
EMT: Emergency Medical Technician
PHQ-9: Patient Health Questionaire-9
GAD-7: Generalized Anxiety Disorder Scale-7
PTCS: Posttraumatic Change Scale
PTSS: Posttraumatic Symptom Scale
SD: Standard deviation
IQR: Inter quartile range
