# Supplementary material for: A cross-sectional study of mental health-, posttraumatic stress symptoms and post exposure changes in Norwegian ambulance personnel
Source: Scand J Trauma Resusc Emerg Med. 2022 Jan 11;30:3. doi: 10.1186/s13049-021-00991-2 (PMC8749923; doi:10.1186/s13049-021-00991-2)
Supplement: Supplementary file 1 — Additional file 1. English version of inex and scales. [file 13049_2021_991_MOESM1_ESM.docx]

**Additional file 1**

**Traumatic events exposure index**

I have experienced the following events as traumatic in my working environment during the last 12 months (Only list one experience per event):

1. Death of young person/ child (˂30 years)

1 Yes, I have experienced the event in the last 12 months, and it was traumatic for me____

2 I have experienced the event in the last 12 months, but it was not traumatic for me____

3 I have not experienced the event in the last 12 months____

2. Serious disease/ traumatic accident in young person/ child (˂30 years)

1 Yes, I have experienced the event in the last 12 months, and it was traumatic for me____

2 I have experienced the event in the last 12 months, but it was not traumatic for me____

3 I have not experienced the event in the last 12 months___

3. Patient with serious injuries

1 Yes, I have experienced the event in the last 12 months, and it was traumatic for me____

2 I have experienced the event in the last 12 months, but it was not traumatic for me____

3 I have not experienced the event in the last 12 months___

4. Suicide

1 Yes, I have experienced the event in the last 12 months, and it was traumatic for me____

2 I have experienced the event in the last 12 months, but it was not traumatic for me____

3 I have not experienced the event in the last 12 months___

5. Death generally

1 Yes, I have experienced the event in the last 12 months, and it was traumatic for me____

2 I have experienced the event in the last 12 months, but it was not traumatic for me____

3 I have not experienced the event in the last 12 months___

6. Inability to help critically ill/ injured patient

1 Yes, I have experienced the event in the last 12 months, and it was traumatic for me____

2 I have experienced the event in the last 12 months, but it was not traumatic for me____

3 I have not experienced the event in the last 12 months___

7. Threats, violence or aggression towards you or colleague

1 Yes, I have experienced the event in the last 12 months, and it was traumatic for me____

2 I have experienced the event in the last 12 months, but it was not traumatic for me____

3 I have not experienced the event in the last 12 months___

8. Threats, violence or aggression towards patients

1 Yes, I have experienced the event in the last 12 months, and it was traumatic for me____

2 I have experienced the event in the last 12 months, but it was not traumatic for me____

3 I have not experienced the event in the last 12 months___

9. Accident in ambulance (An accident is defined as an unplanned event that has caused equipment damage or human injury requiring medical attention (The accident questions do not relate to patient treatment))

1 Yes, I have experienced the event in the last 12 months, and it was traumatic for me____

2 I have experienced the event in the last 12 months, but it was not traumatic for me____

3 I have not experienced the event in the last 12 months___

10. Diagnostic-/ treatment mistake (deviation) causing injury/ clinical deterioration to patient

1 Yes, I have experienced the event in the last 12 months, and it was traumatic for me____

2 I have experienced the event in the last 12 months, but it was not traumatic for me____

3 I have not experienced the event in the last 12 months___

**PHQ-9 (Patient Health Questionnaire)**

How often have you been troubled by the following over the past two weeks:

Not at all 0

Several days 1

More than half the days 2

Nearly every day 3

1. Little interest or pleasure in doing things?

2. Feeling down, depressed, or hopeless?

3. Trouble falling or staying asleep, or sleeping too much?

4. Feeling tired or having little energy?

5. Poor appetite or overeating?

6. Feeling bad about yourself – or that you are a failure or have let yourself or your family down?

7. Trouble concentrating on things, such as reading the newspaper or watching television?

8. Moving or speaking so slowly that other people could have noticed? Or so fidgety or restless that you have been moving a lot more than usual?

9. Thoughts that you would be better off dead, or thoughts of hurting yourself in some way?

**GAD-7 (Generalized Anxiety Disorder) scale**

How often have you been troubled by the following over the past two weeks:

Not at all 0

Several days 1

More than half the days 2

Nearly every day 3

1. Feeling nervous, anxious or on edge?

2. Not being able to stop or control worrying?

3. Worrying too much about different things?

4. Trouble relaxing?

5. Being so restless that it is hard to sit still?

6. Becoming easily annoyed or irritable?

7. Feeling afraid as if something awful might happen?

**PTSS (Posttraumatic symptom scale)**

This section contains 10 questions, and deals with your present well-being. Questions relate to typical reactions that occur as a result of strain or stress. Please mark the number that best describes how you have felt during the last 7 days. Please answer all the questions.

During the last 7 days I have been suffering from:

1. Sleep problems

1_______2_______3_______4_______5_______6_______7_______

Never Always

2. Dreams or nightmares related to experiences that stand out.

1_______2_______3_______4_______5_______6_______7_______

Never Always

3. Depression. I feel dejected/ down-trodden.

1_______2_______3_______4_______5_______6_______7_______

Never Always

4. Jumpiness. I am easily frightened by sudden sounds I hear or sudden movements I see.

1_______2_______3_______4_______5_______6_______7_______

Never Always

5. The need to withdraw from others

1_______2_______3_______4_______5_______6_______7_______

Never Always

6. Irritability, that is, I am easily agitated/ annoyed and angry

1_______2_______3_______4_______5_______6_______7_______

Never Always

7. Frequent mood swings

1_______2_______3_______4_______5_______6_______7_______

Never Always

8. Bad conscience, blame myself, have feelings of guilt

1_______2_______3_______4_______5_______6_______7_______

Never Always

9. Fear of places and situations, which remind me of special circumstances

1_______2_______3_______4_______5_______6_______7_______

Never Always

10. Muscular tension

1_______2_______3_______4_______5_______6_______7_______

Never Always

**PTCS (Posttraumatic change scale)**

To what degree have you experienced changes on a personal level as a consequence of your occupational exposure in the Emergency Medical services?

Answers: 1. Much worse/ -less than before 2. Worse/ less than before 3. Same as before

4. Better/ more than before 5. Much better/ -more than before

1. I feel confident I can handle unexpected situations…..

2. My ability to make my own decisions is…..

3. My ability to manage stress is…..

4. My mental strength is ….

5. My trust in other people is….

6. My ability to be emotionally close to other people is….

7. My contact with other people in general is….

8. My social life is….

9. I am conscious of my priorities in life….

10. I enjoy the “little” moments in life…

11. I live in accordance to my inner values….

12. I appreciate life ….
