# Supplementary material for: A cross-sectional study of mental health-, posttraumatic stress symptoms and post exposure changes in Norwegian ambulance personnel
Source: Scand J Trauma Resusc Emerg Med. 2022 Jan 11;30:3. doi: 10.1186/s13049-021-00991-2 (PMC8749923; doi:10.1186/s13049-021-00991-2)
Supplement: Supplementary file 2 — Additional file 2. Study invitation and consent to participation. [file 13049_2021_991_MOESM2_ESM.pdf]

VIL DU DELTA I FORSKNINGSPROSJEKTET

En studie av psykisk helse, posttraumatiske stress symptomer og emosjonelle endringer blant Norske ambulansearbeidere

FORMÅLET MED PROSJEKTET OG HVORFOR DU BLIR SPURT

Det å arbeide i ambulansetjenesten betyr at man kan bli utsatt for hendelser som oppleves som belastende for den enkelte. Dette kan være alvorlig for den enkelte, men vi vet ikke nok om omfanget av dette. Undersøkelsen skal kartlegge hvordan arbeid i ambulansetjenesten påvirker psykisk helse, og i hvilken grad de rapporterer om stress symptomer og endringer som følge av erfaringer i tjenesten. Du arbeider operativt i ambulansetjenesten i Region Midt Norge, og er blitt identifisert ved at du står på mailliste for denne gruppen.

HVA INNEBÆRER PROSJEKTET FOR DEG?

Du blir bedt om å delta i en spørreundersøkelse. Det tar ca 25 minutter å svare på spørsmålene.

I prosjektet vil vi innhente og registrere opplysninger om deg. Som en del av undersøkelsen skal man også oppgi alder, kjønn, profesjonell bakgrunn, antall års arbeidserfaring og sivil status. Dette i tillegg til spørsmål om spesielle hendelser man kan ha opplevd og direkte spørsmål om psykisk helse og velvære. Du skal ikke oppgi hvilken stasjon eller i hvilket helseforetak du arbeider på. Det gjør at gjenkjenning av identitet til deltaker er svært vanskelig. Prosjektledelse kan heller ikke vite hvem som deltar og hvem som ikke deltar i undersøkelsen.

MULIGE FORDELER OG ULEMPER

Grunnen til at vi gjennomfører denne undersøkelsen er for å kartlegge om temaet for undersøkelsen er en utfordring for dere som arbeider operativt i ambulansetjenesten. Vi tror at en studie fra Midt Norge vil også være representativ for store deler av vår nasjonale tjeneste. Noen av spørsmålene kan sette i gang ubehagelige følelser, hvis man har opplevd sterke hendelser som man blir minnet om. I så fall ber vi deg om å snakke med en kollega, ta i bruke kollegastøtteordningen, henvende deg til en leder du har tillit til eller også å oppsøke fastlege.

FRIVILLIG DELTAKELSE OG MULIGHET FOR Å TREKKE DITT SAMTYKKE

Det er frivillig å delta i denne undersøkelsen. Dersom du ønsker å delta, godkjenner du samtykkeerklæringen på siste side. Undersøkelsen er nettbasert og oppfyller kriterier for GDPR (General Data Protection Regulation). Svarene dine vil bli behandlet strengt konfidensielt og din identitet kan ikke spores.

HVA SKJER MED OPPLYSNINGENE OM DEG?

Opplysningene som registreres om deg skal kun brukes slik som beskrevet under formålet med prosjektet, og planlegges brukt i 2021. Eventuelle utvidelser i bruk og oppbevaringstid kan kun skje etter godkjenning fra REK og andre relevante myndigheter. Du har også rett til å få innsyn i sikkerhetstiltakene ved behandling av

opplysningene. Du kan klage på behandlingen av dine opplysninger til Datatilsynet og institusjonen sitt personvernombud.

Alle opplysningene vil bli behandlet uten navn og fødselsnummer eller andre direkte gjenkjennende opplysninger (=kodede opplysninger).

Publisering av resultater er en nødvendig del av forskningsprosessen. All publisering skal gjøres slik at enkeltdeltakere ikke skal kunne gjenkjennes, men vi plikter å informere deg om at vi ikke kan utelukke at det kan skje, selv om sannsynligheten er svært lite for dette.

Opplysningene om deg vil bli oppbevart i fem år etter prosjektslutt av kontrollhensyn.

FORSIKRING

Du er forsikret som arbeidstaker i Helseforetaket.

ØKONOMI

Prosjektet og deltakere i spørreundersøkelsen mottar ingen økonomiske motytelser for dette prosjektet.

GODKJENNINGER

Regional komité for medisinsk og helsefaglig forskningsetikk har gjort en forskningsetisk vurdering og godkjent prosjektet: 199839

St Olavs Hospital HF og prosjektleder Oddvar Uleberg er ansvarlig for personvernet i prosjektet.

Vi behandler opplysningene basert på behandlingsgrunnlag, etter konferanse med PVO ved St. Olavs Hospital HF

KONTAKTOPPLYSNINGER

Dersom du har spørsmål til prosjektet kan du kontakte: Bjørn Ole Reid, Akuttmedisinsk Fagavdeling/ St Olavs Hospital HF, Tlf: 40488893, bjorn.ole.reid@stolav.no

Dersom du har spørsmål om personvernet i prosjektet, kan du kontakte personvernombudet ved St Olavs Hospital HF: Sevian.Stenvig@stolav.no

Datatilsynets veiledningstelefon er: 22396900

Delta i undersøkelsen

Takk for at du vil ta deg tid til å besvare undersøkelsen. Din tilbakemelding er svært viktig for oss.

Ved å krysse av for at «jeg ønsker å delta» gir du tillatelse til at din besvarelse brukes til en studie om psykisk helse, stress symptomer og emosjonell utvikling i ambulansetjenesten.

JEG SAMTYKKER TIL Å DELTA I PROSJEKTET OG TIL AT MINE PERSONOPPLYSNINGER BRUKES SLIK DET ER BESKREVET

Ja, jeg ønsker å delta_____

Nei, jeg ønsker ikke å delta_____
